# Predictive Value of the Serum CRP/Prealbumin Ratio for Sepsis in Pregnant and Postpartum Women

**DOI:** 10.1155/jp/8227735

**Published:** 2026-08-31

**Authors:** Yuan-Qin Huang, Qi Wang, Dou-Dou Xiang, Quan Gan

**Affiliations:** ^1^ Department of Intensive Care Unit, Maternal and Child Health Hospital of Hubei Province, Wuhan, China; ^2^ Division of Gastroenterology and Hepatology, Renji Hospital, Shanghai Jiao-Tong University, Shanghai, China, renji.com; ^3^ Department of Laboratory Section, Maternal and Child Health Hospital of Hubei Province, Wuhan, China

**Keywords:** C-reactive protein, prealbumin, procalcitonin, sepsis, serum amyloid A protein

## Abstract

**Purpose:**

To monitor serum C‐reactive protein (CRP) and prealbumin (PA) levels and assess the clinical value of their ratio (CRP/PA) for predicting perioperative sepsis in puerperal women.

**Methods:**

We prospectively enrolled 142 pregnant (*n* = 77) and postpartum (*n* = 65) women with suspected sepsis who were treated at the Maternal and Child Health Hospital of Hubei Province between August 2021 and March 2023. Among them, 110 and 32 patients who were admitted to the Department of Internal Medicine and adult intensive care unit (ICU) were assigned to the mild and severe sepsis groups, respectively. For each patient, the respiratory rate, heart rate, and blood pressure were recorded; blood samples were collected on the day of admission and after 72 h of treatment; serum CRP and PA levels were determined; and their CRP/PA ratio was calculated. Receiver operating characteristic (ROC) curves were used to evaluate the ability of the various indicators to predict septic shock.

**Results:**

The serum CRP/PA ratio, procalcitonin (PCT) level, and serum amyloid A (SAA) protein level were significantly greater in the severe sepsis group than in the mild sepsis group (*p* < 0.05). In the severe sepsis group, the serum CRP/PA ratio and PCT level were significantly greater after 72 h of treatment than on the day of diagnosis (*p* < 0.05).

**Conclusion:**

The CRP/PA ratio has a predictive value, with an increased ratio indicating an increased risk of septic shock.

## 1. Introduction

Maternal sepsis complicates approximately 1–2 of every 1000 pregnancies worldwide, with a pooled incidence of 13.2 per 10,000 women, and is the third leading direct cause of maternal mortality worldwide [1]. The repercussions extend to the fetus and newborn, as inflammatory dysregulation that accompanies maternal sepsis is linked to increased risks of preterm birth, stillbirth, and early‐onset neonatal sepsis. Consequently, timely recognition of impending septic shock during pregnancy or the puerperium is critical for the survival of both the mother and child [2]. At present, the definition of sepsis in the relevant guidelines does not account for the physiological changes that occur during normal pregnancy. The use of nonpregnancy‐related indicators may lead to the over‐ or underdiagnosis of pregnant or puerperal women, and previously healthy pregnant women need to pay attention to the symptoms of sepsis to achieve timely diagnosis and treatment. Most reports of sepsis in these women do not specifically mention pathogenic microorganisms, with the causative organism being identified through clinical laboratory tests in only 64% of cases in the United Kingdom obstetric testing system. Clinicians identify the source of sepsis in only 74% of infected women, and no causative organism or source of sepsis has been identified in 16% of infected pregnant women [3].

Several single‐analyte tests, such as those measuring PCT, SAA, interleukin‐6 (IL‐6), and CRP levels, are incorporated into routine obstetric practice; however, none achieve the combination of speed, sensitivity, and obstetric‐specific specificity that is required for early sepsis escalation. All four markers increase in a broad spectrum of inflammatory states (surgery, trauma, and autoimmune flare), which blunts their diagnostic precision and prognostic calibration in pregnant women [4]. The concentration of CRP, an IL‐6‐inducible positive acute‐phase protein, surges within hours of tissue injury, whereas PA is a negative acute‐phase reactant protein whose concentration mirrors the hepatic synthetic response and short‐term protein nutritional status. Mounting evidence has shown that increasing CRP levels and decreasing PA levels track sepsis severity in obstetric cohorts and that compared with either marker alone or with PCT and SAA levels, the CRP/PA ratio enhances prognostic discrimination. Conceptually, this ratio couples host injury intensity (inflammation) with the simultaneous depletion of hepatic protein reserves (nutrition/immune competence), yielding a composite signal of a deteriorating physiological reserve.

Despite its mechanistic appeal, the CRP/PA ratio has never been prospectively evaluated as a bedside predictor of septic shock among pregnant and postpartum women [5]. Therefore, the aim of the present study was to characterize the dynamic changes in the CRP/PA ratio in patients with obstetric sepsis and determine its prognostic accuracy relative to that of the PCT and SAA levels for the early identification of women at risk for progression to septic shock.

## 2. Results

### 2.1. Baseline Characteristics

No significant differences were observed in age, body temperature, respiration, heart rate, white blood cell count, platelet count, albumin level, prealbumin level, total bilirubin level, or creatinine level between the mild and severe infection groups on the day of diagnosis (*p* > 0.05). No significant differences were observed in body temperature, respiration, heart rate, white blood cell count, albumin level, or total bilirubin level between the two groups after 72 h of treatment (*p* > 0.05); however, in the severe infection group, the platelet count and prealbumin level were significantly lower and the creatinine level was significantly higher than in the mild infection group (*p* < 0.05).

### 2.2. Dynamic Changes in Biomarkers

Serum CRP levels did not significantly differ between the two groups on the day of diagnosis (*p* > 0.05); however, the serum CRP/PA ratio, PCT and SAA levels, and Acute Physiology and Chronic Health Evaluation (APACHE II) scores were significantly higher in the severe infection group than in the internal medicine group (*p* < 0.05; Table 1).

**Table 1:** Comparison of indicators on the day of diagnosis in the two groups.

Constituencies	CRP (mean ± SD, mg/L)	PCT (mean ± SD, *μ*g/L)	CRP/PA	SAA (mean ± SD, mg/L)	APACHE II (mean ± SD)
Mild infection group	88.76 ± 39.21	1.83 ± 0.55	0.99 ± 0.53	245 ± 49	20.47 ± 7.91
Severe infection group	98.19 ± 33.18	3.23 ± 1.89	2.01 ± 1.47	289 ± 27	26.69 ± 8.59
*p* value	> 0.05	< 0.05	< 0.05	< 0.05	< 0.05

*Note:* The CRP levels and APACHE II scores were normally distributed, and the Student′s *t*‐test was used for comparisons between groups. The PCT level and CRP/PA ratio were nonnormally distributed, and the Mann–Whitney *U* test was used for comparisons between groups.

Comparisons between the serum CRP levels, CRP/PA ratios, PCT and SAA levels and APACHE II scores on the day of diagnosis and after 72 h of treatment in the severe infection group showed that the serum CRP/PA ratio, PCT level, and APACHE II score were significantly greater at 72 h than on the day of diagnosis (*p* < 0.05); however, there were no significant differences in the serum CRP levels between the two groups at the two time points (*p* > 0.05) (see Table 2).

**Table 2:** Comparison of indexes at different time points in the severe infection group (*n* = 32).

Observation time	CRP (mean ± SD, mg/L)	PCT (mean ± SD, *μ*g/L)	CRP/PA	SAA (mean ± SD, mg/L)	APACHE II (mean ± SD)
On the day of diagnosis	98.19 ± 33.18	3.23 ± 1.89	2.01 ± 1.47	289 ± 27	26.69 ± 8.59
After 72 h of treatment	103.73 ± 29.74	5.5 ± 1.19	3.68 ± 2.15	193 ± 97	31.27 ± 5.38
*p* value	> 0.05	< 0.05	< 0.05	—	< 0.05

Note: The CRP levels and APACHE II scores were normally distributed, and the Student′s *t*‐test was used for comparisons between groups. The PCT level and CRP/PA ratio were nonnormally distributed, and the Mann–Whitney *U* test was used for comparisons between groups.

Notably, although serum CRP levels were not significantly different between the groups at diagnosis (*p* > 0.05), the CRP/PA ratio was significantly elevated in the severe sepsis group. This finding suggests that the ratio, which integrates the opposing dynamics of a positive (CRP) and a negative (PA) acute‐phase reactant protein, provides a better discriminative signal of disease severity than the CRP level alone does.

### 2.3. Predictive Performance of Biomarkers

**Figure 1:**
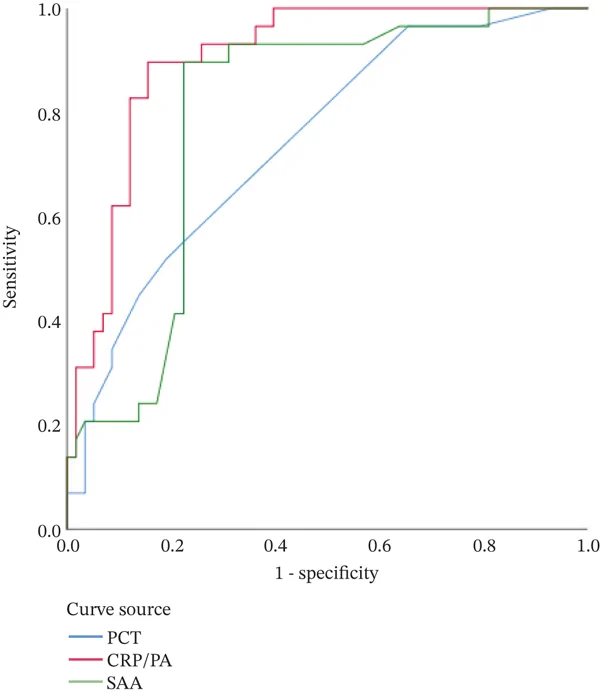
ROC curves of serum CRP/PA, PCT, and SAA for predicting sepsis shock.

The severity of each patient′s condition was assessed by using a ROC curve for each indicator. The area under the ROC curve of the CRP/PA ratio was the largest (0.766), indicating that this indicator was the best at predicting septic shock (Table 3 and Figure 1). A CRP/PA ratio cutoff of 2.4 yielded the best sensitivity (77.5%) and specificity (72.2%), with an accuracy of 73.7% for predicting septic shock.

**Table 3:** Area under the curve of serum CRP/PA, PCT, and SAA in predicting sepsis shock.

Diagnostic indicators	Area under the ROC curve	95% CI		*p*
		Upper limit	Lower limit	
CRP/PA	0.766	0.686	0.846	< 0.05
PCT	0.656	0.552	0.759	< 0.05
SAA	0.554	0.492	0.613	< 0.05

## 3. Methods

A total of 142 patients were enrolled and subsequently classified into a mild sepsis group (*n* = 110), which included those who were admitted to the Department of Internal Medicine, and a severe sepsis group (*n* = 32), which included those who were admitted to the adult intensive care unit (ICU). The study cohort included 77 pregnant women and 65 women in the puerperium period, who were aged 19–47 years, with an average age of 31.27 ± 5.82 years. The sample size was based on a convenience sample of eligible patients who presented during the study period. A post hoc power analysis was performed, which indicated that the study had a power of > 80% to detect a significant difference in the primary biomarker (CRP/PA ratio) between the groups. The main sites of sepsis were as follows: the respiratory system, 23 cases; the genitourinary system, 61 cases; the digestive system, 13 cases; skin and soft tissue, 9 cases; blood, 10 cases; unknown, 23 cases; and infectious abortion, 2 cases. There was one case of mortality.

The inclusion criteria were as follows: (1) Patients who met the diagnostic criteria of the “Guidelines for the Management of Sepsis in Pregnancy and Puerperium” [6] issued by the Society for Maternal–Fetal Medicine (SMFM) in 2019; (2) patients with suspected sepsis were evaluated according to the sequential organ failure assessment (SOFA) score (a: systolic blood pressure < 90 mmHg (1 mmHg = 0.133 kPa); b: respiratory rate ≥ 25 breaths/min; c: altered mental status); patients with a score ≥2 were admitted to the adult ICU; otherwise, they were admitted to the adult internal medicine department. The exclusion criteria were as follows: (1) death or discharge within 24 h of ICU admission; (2) automatic discharge from the hospital or abandonment of treatment; and (3) leukopenia, agranulocytosis, AIDS, or other immunosuppressive conditions, such as ongoing long‐term hormone therapy and past bone marrow transplantation or solid organ transplantation.

After the patients were diagnosed with sepsis, they were treated in accordance with the standards of “Improving the Diagnosis and Treatment of Sepsis in Pregnancy and Puerperium” (2020). The age, underlying diseases or status, main diagnosis, and outcome of patients who met the criteria were recorded. Basic physiological indicators, such as the body temperature, respiratory rate, and heart rate, were recorded on the day of diagnosis and after 72 h of treatment, and APACHE II scores were calculated. Peripheral blood was collected on the day of diagnosis and after 72 h of treatment, and serum CRP, PA, PCT, and SAA levels were measured. Serum CRP levels were determined by latex‐enhanced immunoturbidimetry on a Mindray BC‐7500 [NR] CS analyzer. PA levels were determined by scattered immunoturbidimetry on a Roche Cobas 702 analyzer. Serum PCT levels were determined by a chemiluminescence particle immunoassay on an Abbott Architect i2000 analyzer. SAA levels were determined by latex‐enhanced immunoscattering turbidimetry on a Mindray C‐7500 [NR] CS analyzer.

## 4. Statistical Analysis

Data normality was assessed via the Shapiro–Wilk test. Continuous data with a normal distribution are presented as the mean ± standard deviation (SD) and were compared by using Student′s t test. Nonnormally distributed data are presented as medians (interquartile ranges) and were compared by using the Mann–Whitney *U* test. Categorical data were compared by using the chi‐square test. Appropriate corrections were considered for multiple comparisons between the groups at different time points. There were no missing data for the primary biomarkers analyzed. All the statistical analyses were performed by using the SPSS software (Version 26.0; IBM Corp., Armonk, New York, United States).

## 5. Conclusion

This prospective study is the first to systematically validate the core value of the CRP/PA ratio for the early prediction of progression to septic shock in a high‐risk obstetric population with sepsis [7, 8]. Our primary finding was that the CRP/PA ratio demonstrated an early warning capability during sepsis. On the day of diagnosis, although the absolute level of CRP, which is the traditional marker of the intensity of the systemic inflammatory response, was not significantly different between the mild and severe sepsis patient groups [9], the CRP/PA ratio was highly significantly different. This finding indicates that before a patient manifests clear clinical signs of organ failure or hemodynamic instability, this ratio can be used to detect early imbalances in internal physiological homeostasis, warning of the high risk of deterioration from a controllable systemic inflammatory response syndrome to uncontrolled septic shock.

Second, this study is the first to systematically delineate the dynamic evolution trajectory of the CRP/PA ratio and its profound prognostic implications in an obstetric sepsis population. We observed that in the severe group of patients, compared with that on the day of diagnosis, the CRP/PA ratio at 72 h of treatment nearly doubled. This dynamic pattern strongly suggests that the initial anti‐infective and supportive therapy failed to effectively halt the pathophysiological process of the disease, creating a vicious positive feedback loop between ongoing inflammatory injury and the depletion of the host′s physiological reserves. Conversely, in the mild group, this ratio decreased synchronously with clinical improvement. Therefore, the dynamic trend of the CRP/PA ratio provides critical evidence‐based guidance for clinical adjustments to treatment regimens.

Third, through rigorous ROC curve analysis, we established a precise quantitative threshold for clinical risk stratification using the CRP/PA ratio. The results of this study revealed that a CRP/PA ratio > 2.4 yielded optimal discriminatory performance for predicting septic shock, with a sensitivity of 77.5%, a specificity of 72.2%, and an area under the curve (AUC) of 0.766. This predictive performance is significantly superior to that of commonly used single inflammatory markers, such as procalcitonin and serum amyloid A, and surpasses even that of the comprehensive APACHE II score. This clear, easily calculable cutoff value translates a complex pathophysiological process into a decision‐making tool applicable at the bedside.

The clinical predictive performance of the CRP/PA ratio is rooted in its integration and quantification of the core pathophysiological mechanisms of sepsis—the dual crises of an excessive inflammatory response and depletion of the host physiological reserve. The modern definition of sepsis emphasizes that it is a life‐threatening organ dysfunction caused by a dysregulated host response to infection. This definition inherently encompasses the following two interrelated but opposing pathological processes: (1) the excessive, damaging systemic inflammatory response, and (2) the physiological reserve exhaustion and organ function decompensation resulting from failed compensatory mechanisms [10]. The CRP/PA ratio increases rapidly within hours of infection or tissue injury. Therefore, the serum CRP concentration is a sensitive indicator of the intensity of the systemic inflammatory response. However, the limitation of using CRP as a single marker is that it measures only the inflammatory load. A markedly elevated CRP level may be observed in a patient with robust physiological reserves who remains compensated and relatively stable; conversely, it may also be observed in a critically ill patient on the brink of collapse because of exhausted reserves. On the basis of the CRP level alone, clinicians struggle to distinguish between these two prognostically distinct scenarios. Owing to its half‐life of approximately 1.9 days, PA has traditionally been used as a sensitive indicator for assessing the short‐term nutritional status and protein synthesis rate. However, in severe stress states, such as sepsis, a decrease in the PA level is highly pathophysiological [11]. In fact, PA is classified as a negative acute‐phase reactant protein [12]. This term indicates that upon severe systemic inflammation, in contrast to the increase in CRP synthesis, the synthesis of PA is actively and significantly suppressed by the body. This suppression is a core manifestation of the body′s strategic adjustment in critical moments. The mechanisms involved span multiple levels; at the transcriptional level, predominant proinflammatory cytokines, such as interleukin‐1*β* (IL‐1*β*), IL‐6, and tumor necrosis factor‐alpha (TNF‐*α*), directly downregulate the transcription of the PA gene in hepatocytes. The liver reprioritizes limited transcription and translation resources for the synthesis of acute‐phase defense proteins, such as CRP, fibrinogen, and complement, which are directly involved in fighting infection and in tissue repair. In terms of metabolic substrate competition, the sepsis‐induced hypercatabolic state leads to massive breakdown of skeletal muscle protein to release amino acids. However, under the drive of inflammatory signals, these raw materials are preferentially directed toward two urgent pathways—one for the synthesis of the aforementioned acute‐phase proteins and the other for gluconeogenesis to supply energy to key organs in a hypermetabolic state. The synthesis of proteins such as PA, which are primarily involved in maintaining homeostasis, is temporarily inhibited. Furthermore, systemic inflammatory response syndrome is often accompanied by capillary leakage, leading to the loss of PA from the intravascular space into the interstitial space, which further reduces its serum concentration. Therefore, in the context of sepsis, a rapid decrease in the PA level is a composite signal that integrates inflammation‐mediated suppression of hepatic synthetic function, competitive consumption of synthetic substrates, and pathological expansion of the intravascular distribution volume. The decrease in the PA level is far more than an indicator of insufficient intake; it profoundly signals the rapid and ongoing consumption of the physiological and metabolic capital that the host uses to maintain the internal environment, support tissue repair, and maintain immune function. A decreasing PA level acts as an early warning for the body, indicating that its capacity to cope with the ongoing inflammatory crisis is near exhaustion. This deep understanding of the opposing roles played by CRP and PA in the aforementioned pathophysiological processes reveals the extraordinary value of the CRP/PA ratio [13]. It is essentially a dynamic inflammation‐reserve balance index. The numerator of this ratio monitors the intensity of the inflammatory attack in real time, whereas its denominator simultaneously monitors the consumption rate of the physiological defense reserve. The continuous change in the ratio dynamically quantifies the net balance between the disease injury expenditure and host repair income. When the CRP/PA ratio continues to increase, it conveys a clear danger signal, indicating that the pace of inflammatory injury has exceeded the limits of the body′s repair and compensatory capacity, and the physiological balance of the entire system is deteriorating, sliding toward decompensation. This description perfectly explains the key observations of this study, namely, why on the day of diagnosis, when a single indicator could not yet distinguish severity, the CRP/PA ratio could already sensitively identify the high‐risk group and why, during treatment, the progressive worsening of the ratio in the severe group patients accurately predicted their nonresponse to current therapy and impending shock.

Approaches that combine inflammatory markers with nutritional/synthetic function markers to enhance prognosis have been validated in multiple medical fields. Bozkurt [14] reported that the CRP/albumin ratio was more sensitive than the CRP level alone in distinguishing disease activity in patients with uveitis. Similarly, this ratio has been shown to be valuable for determining the prognosis of conditions such as acute pancreatitis [3], various malignancies, and acute exacerbations of chronic obstructive pulmonary disease. These studies collectively corroborate a universal biological principle that the severity and outcome of a disease depend not only on the intensity of the damaging factors but also, equally, on the host′s intrinsic potential to maintain homeostasis and repair damage. Any composite indicator that can simultaneously reflect both of these aspects is typically more discriminatory than a single‐dimensional indicator. However, the prominent contribution of this study lies in its being the first to prospectively and systematically apply this validated biological effect on the kinetics of traditional biomarkers. Within this complex context, the CRP/PA ratio potentially has specific advantages. First, as a ratio, it possesses a certain correction potential. Pregnancy‐induced blood volume expands this principle to the clinically specific scenario of obstetric sepsis, which has an extremely unique physiological background, and confirms its unique potential advantages in this setting. The maternal body undergoes a series of profound physiological adaptations during pregnancy, and these changes pose special challenges for the diagnosis and management of sepsis: A 40%–50% increase in the blood volume during pregnancy causes hemodilution, potentially leading to the underestimation of the absolute values of inflammatory markers, including CRP; simultaneously, the mild physiological inflammatory state present in the mid‐to‐late stages of pregnancy elevates the normal reference ranges for indicators such as CRP, increasing the difficulty of distinguishing physiological from pathological states. The diagnostic criteria of general sepsis are not designed for pregnant women, and some of the parameters included undergo physiological changes during pregnancy; thus, direct application may lead to misjudgment. To maintain immune tolerance to the fetus, the maternal immune system shifts overall toward a Th2 bias [15], which may alter its overall response pattern to infection; ions dilute both CRP and PA simultaneously, but the ratio calculation may, to some extent, offset the impact of this dilution on the interpretation of absolute values. More importantly, the decrease in PA levels primarily reflects reduced synthesis and reserve depletion in the liver under severe inflammatory stress rather than a simple dilution effect. Therefore, even if a pregnant woman′s absolute CRP value, owing to dilution, does not reach the warning threshold for nonpregnant people, a sharp decrease in her PA level, which is caused by a severe pathological process and leads to a significantly elevated CRP/PA ratio, strongly suggests that the current level of inflammation poses a serious threat relative to her limited physiological reserves, indicating a greater clinical warning. Second, PA, as an indicator that is closely linked to nutritional metabolism and hepatic synthetic function, is directly associated with fetal growth and development and the mother′s ability to cope with long‐term stress. In obstetric clinical practice, monitoring the mother′s anabolic reserves is particularly important. Consequently, the CRP/PA ratio not only reflects the acute severity of sepsis but also indirectly reflects the long‐term risk that the disease poses to the overall prognosis of both the mother and child. In summary, this study not only validated the effectiveness and superiority of the CRP/PA ratio in an obstetric sepsis population but also suggested that it may be an early warning tool that is particularly well adapted to the complex physiological changes associated with pregnancy. This is precisely the key link in improving the diagnosis and treatment of obstetric sepsis.

On the basis of the above findings, the CRP/PA ratio can be integrated into a multilevel clinical decision support framework; for pregnant and postpartum women with suspected infection, the PA level should be tested simultaneously with the CRP level, and the ratio should be calculated. A ratio > 2.4 should serve as an objective trigger to activate a sepsis warning and management bundle to ensure that the patient enters a higher level of the monitoring protocol and to prompt an active search for the infection source. After diagnosis, this ratio should be dynamically monitored every 24–48 h. Its trend is among the core indicators for assessing the effectiveness of the initial anti‐infective treatment regimen. If the ratio continues to increase 48–72 h after treatment initiation, a mandatory reevaluation of the treatment plan must be initiated. This ratio can aid in optimizing the allocation of critical medical resources. Moreover, this study provides a new biomarker endpoint and target for future research on goal‐directed individualized treatment. We are fully aware of the limitations of this study. First, it is a single‐center study with a modest sample size, which may limit the generalizability of the findings. Second, we did not stratify patients by gestational age, which could influence biomarker levels. Third, although we used the SOFA score and ICU admission for classification, other (unmeasured) confounding factors (e.g., specific pathogens and comorbidities) could influence outcomes [16]. Future large‐scale, multicenter studies are needed to validate these findings.

This study provides prospective evidence that the serum CRP/PA ratio is a powerful composite biomarker capable of providing an early, dynamic, and high‐precision warning of physiological reserve depletion and the risk of progression to septic shock in pregnant and postpartum women with sepsis [17]. This discovery not only provides a novel and powerful quantitative tool for clinical decision‐making in obstetric critical care but also deepens our understanding of the nature of sepsis during pregnancy—it is not merely an overreaction of the immune system but a collapse of the host’s overall physiological balance [18]. The value of this study lies in its direct potential for clinical translation and the research paradigm it exemplifies—deepening the understanding of disease by integrating multidimensional pathophysiological information—which has profound implications for the advancement of obstetric critical care medicine.

## Author Contributions

All the authors contributed to the conception and design of the study. Material preparation, data collection, and analyses were performed by Yuan‐Qin Huang, Qi Wang, and Dou‐Dou Xiang. The first draft of the manuscript was written by Yuan‐Qin Huang, and all the authors commented on the previous versions of the manuscript.

## Funding

No funding was received for this manuscript.

## Disclosure

All the authors have read and approved the final manuscript.

## Ethics Statement

This study was conducted in accordance with the principles of the Declaration of Helsinki. Approval was granted by the Ethics Committee of the Maternal and Child Health Hospital of Hubei Province (Approval No. [2023] IEC [103]).

## Consent

Informed consent was obtained from all the participants included in this study. The authors affirm that the research participants provided informed consent for the publication of the data in Tables 1, 2, and 3 and Figure 1.

## Conflicts of Interest

The authors declare no conflicts of interest.

## Data Availability

Data sharing is not applicable to this article, as no datasets were generated or analyzed in the current study.

## References

[1] Yu C. , Lv H. , Fang W. , Zhang X. , and Huang L. , Global Incidence of Maternal Sepsis: A Systematic Review and Meta-Analysis, Journal of Gynecology Obstetrics and Human Reproduction. (2025) 54, no. 5, 102940, 10.1016/j.jogoh.2025.102940. 40056980

[2] Newton E. R. , O′Neil E. , Spieker A. , Hebbar L. , and Prins S. A. , Maternal Early Warning Criteria (MEWS) Reduce Time to Sepsis Recognition in Hospitalized Pregnant Women, American Journal of Perinatology. (2021) 38, no. 6, 546–552.

[3] Piñerúa-Gonsálvez J. F. , Ruiz Rebollo M. L. , Zambrano-Infantino R. D. C. , Rizzo-Rodríguez M. A. , and Fernández-Salazar L. , Value of CRP/Albumin Ratio As a Prognostic Marker of Acute Pancreatitis: A Retrospective Study, Revista Española de Enfermedades Digestivas. (2023) 115, 707–712, 10.17235/reed.2023.9345/2022. 37539554

[4] Plante L. A. , Juusela J. L. , Paisner N. D. , and Newton E. R. , Diagnostic Thresholds of Traditional Biomarkers for Maternal Sepsis Are Invalidated by Physiologic Pregnancy Adaptations, American Journal of Obstetrics and Gynecology. (2021) 225, no. 1, 61.e1–61.e9.

[5] Sprung C. L. , Bauer K. A. , and Reinhart K. , Multicenter Evaluation of Procalcitonin, C-Reactive Protein, and Clinical Scores in Sepsis: The Sepsis Biomarker Mapping Analysis (SEPTIMAP) Study, Critical Care Medicine. (2021) 49, no. 12, 2048–2057.

[6] Plante L. A. , Pacheco L. D. , and Louis J. M. , SMFM Consult Series #47: Sepsis During Pregnancy and the Puerperium, Am J Obstet Gynecol. (2019) 220, no. 4, B2–B10, 10.1016/j.ajog.2019.01.216.Epub. 30684460

[7] Song Y. , Zhong H. , Li L. , Yin M. , Yin Y. , Guo X. , Zhang B. , and Liu W. , Dynamic Monitoring of Immune Function Indexes in COVID-19 Patients, Aging. (2020) 12, no. 24, 24596–24603, 10.18632/aging.202362. 33361525 PMC7803549

[8] Bi J. , Zhang Y. , Zhang J. , Han Q. , Ji C. , and Zhen W. , Effects of Lymphocyte, C-Reactive Protein and Prealbumin Levels on Clinical Typing and Course of Disease in Children Infected With Novel Coronavirus, Pakistan Journal of Medical Sciences. (2022) 38, no. 5, 1250–1254, 10.12669/pjms.38.5.5286. 35799744 PMC9247754

[9] Kamal D. E. , Zaghlol R. S. , Hussien M. H. S. , and Makarm W. K. , Utility of Neutrophil/Albumin Ratio and C-Reactive Protein/Albumin Ratio As Novel Inflammatory Markers in Behcet′s Disease, Reumatología Clínica. (2023) 19, no. 4, 188–196, 10.1016/j.reumae.2023.03.005.PMID. 37061280

[10] Sneh A. , Pawan T. , Randeep G. , Anant M. , Mani K. , Hadda V. , and Madan K. , Acute Phase Proteins As Predictors of Survival in Patients With Acute Exacerbation of Chronic Obstructive Pulmonary Disease Requiring Mechanical Ventilation, Journal of Chronic Obstructive Pulmonary Disease. (2020) 17, no. 1, 22–28, 10.1080/15412555.2019.1698019.Epub. 31820666

[11] Rebnord I. K. , Bruk av CRP på legevakt, Tidsskrift for Den norske legeforening. (2017) 137, no. 20, 10.4045/tidsskr.17.0766, 29094578. 29094578

[12] Zhang C. , Liu P. , Xia K. , Fang H. , Jiang M. , Xie Q. , Yu Z. , and Yang T. , Association of Serum Prealbumin With Angiographic Severity in Patients With Acute Coronary Syndrome, Medical Science Monitor. (2017) 21, no. 23, 4041–4049, 10.12659/msm.902348. PMC5574376 28827514

[13] Ji W. , Liu Z. , and Lin T. , Diagnostic Value of Albumin/Fibrinogen Ratio and C-Reactive Protein/Albumin/Globulin Ratio for Periprosthetic Joint Infection: A Retrospective Study, PeerJ. (2023) 15, no. 11, e16662, 10.7717/peerj.16662.PMID. PMC10726739 38111666

[14] Bozkurt E. , Muhafiz E. , Sengul D. , Uçak T. , and Atum M. , Can the CRP/Albumin Ratio Be Used As a New Indicator of Activation in Patients With Uveitis?, Ocular Immunology and Inflammation. (2021) 29, no. 5, 1017–1022, 10.1080/09273948.2020.1714061.Epub. 32125910

[15] Mor G. and Cardenas I. , The Immune System in Pregnancy: A Unique Complexity, American Journal of Reproductive Immunology. (2010) 63, no. 6, 425–433, 10.1111/j.1600-0897.2010.00836.x. 20367629 PMC3025805

[16] Mocellin M. C. , Pastore e Silva J. D. , Camargo Cde Q. , Fabre M. E. , Gevaerd S. , Naliwaiko K. , Moreno Y. M. , Nunes E. A. , and Trindade E. B. , Fish Oil Decreases C-Reactive Protein/Albumin Ratio Improving Nutritional Prognosis and plasma Fatty Acid Profile in Colorectal Cancer Patients, Lipids. (2013) 48, no. 9, 879–888, 10.1007/s11745-013-3816-0, 23888317. 23888317

[17] Yang X. , Wang Y. , Zhao S. , Huang X. , Tian B. , Yu R. , and Ding Q. , Clinical Characteristics and Prognosis of *Klebsiella pneumoniae* Meningitis in Adults, Heliyon. (2024) 10, no. 7, e28010, 10.1016/j.heliyon.2024.e28010.PMID. 38601552 PMC11004708

[18] Wu M. , Guo J. , Guo L. , and Zuo Q. , The C-Reactive Protein/Albumin Ratio Predicts Overall Survival of Patients With Advanced Pancreatic Cancer, Tumor Biology. (2016) 37, no. 9, 12525–12533, 10.1007/s13277-016-5122-y. 27344157 PMC5080377

